# Group F Streptococcal Bacteremia Complicating a Bartholin's Abscess

**DOI:** 10.1155/S1064744901000102

**Published:** 2001

**Authors:** Alan J. DeAngelo, David P. Dooley, Peter J. Skidmore, Craig T. Kopecky

**Affiliations:** 1 Department of Medicine Brooke Army Medical Center Texas USA; 2 Infectious Disease Service Brooke Army Medical Center Fort Sam Houston Texas USA; 3 Brooke Army Medical Center Attn. MCHE-MD 3851 Roger Brooke Drive Fort Sam Houston TX 78234-6200 USA

## Abstract

*Background:* Group F streptococci are Gram-positive cocci typically isolated from wound infections and abscesses. Bacteremia with group F streptococcus is uncommon, and the lower gynecologic tract has not been reported as a source. We report a case of a Bartholin's abscess leading to group F streptococcal bacteremia.

*Case:* A 31-year-old female noted fever and rigors 30 min after manipulation of a 3-day-old vulvar abscess. An empty Bartholin's gland abscess was found on examination, and blood cultures grew β-hemolytic group F streptococci. The patient was treated with ampicillin/sulbactam, symptoms improved, and follow-up blood cultures revealed no growth.

*Conclusion:* Group F streptococci are known to inhabit various body sites and have a predilection for forming abscesses; however, bacteremia is infrequent. They have occasionally been identified in true infections of the genitourinary tract but only very rarely in Bartholin's abscesses. This case of group F streptococcal bacteremia following self-drainage of a Bartholin's abscess constitutes the first such description in the medical literature.


Infect Dis Obstet Gynecol 2001;9:55–57

### Group F streptococcal bacteremia complicating a Bartholin's abscess

**Alan J. DeAngelo[1], David P. Dooley[2], Peter J. Skidmore[2] and Craig T. Kopecky[1]**

[1]*Department of Medicine, Brooke Army Medical Center*
[2]*Infectious Disease Service, Brooke Army Medical Center, Fort Sam Houston, Texas, USA*

***Background:*** Group F streptococci are Gram-positive cocci typically isolated from wound infections and abscesses. Bacteremia with group F streptococcus is uncommon, and the lower gynecologic tract has not been reported as a source. We report a case of a Bartholin's abscess leading to group F streptococcal bacteremia.
***Case:*** A 31-year-old female noted fever and rigors 30 min after manipulation of a 3-day-old vulvar abscess. An empty Bartholin's gland abscess was found on examination, and blood cultures grew β-hemolytic group F streptococci. The patient was treated with ampicillin/sulbactam, symptoms improved, and follow-up blood cultures revealed no growth.
***Conclusion:*** Group F streptococci are known to inhabit various body sites and have a predilection for forming abscesses; however, bacteremia is infrequent. They have occasionally been identified in true infections of the genitourinary tract but only very rarely in Bartholin's abscesses. This case of group F streptococcal bacteremia following self-drainage of a Bartholin's abscess constitutes the first such description in the medical literature.

Key words: VULVAR INFECTION; *STREPTOCOCCUS MILLERI*

The group F streptococci are Gram-positive, facultatively anaerobic cocci that naturally inhabit the genitourinary tract. Group F streptococci are known for their propensity to cause tissue abscesses but are uncommon isolates in bacteremia. These organisms very rarely cause gynecologic infections. We report a case of group F streptococcal bacteremia following self-drainage of a Bartholin's abscess.

### CASE REPORT

A 31-year-old female had first noted the appearance of a painful vulvar mass 3 days prior to presentation. Thirty minutes after vigorously expressing purulent, bloody, maladorous fluid from the mass, she noted the onset of fever and rigors and presented to the Emergency Department. The patient reported her last menstrual period to be 3 weeks prior to presentation and denied use of tampons. On arrival her temperature was recorded at 40.5°C, the blood pressure was 104/60 mmHg, and the pulse was 116 bpm. Physical examination revealed erythema and edema of the right labium majus, without evidence of a sinus tract or purulent drainage; therefore, cultures could not be obtained, and no surgical procedure was performed. No other lesions were noted, and the remainder of the pelvic examination was normal. Urine and vaginal cultures were negative, and the

The opinions or assertions contained herein are the private views of the authors and are not to be construed as reflecting the views of the Departments of the Army or Defense.
Correspondence to: Alan J. DeAngelo, MD, Brooke Army Medical Center, Attn. MCHE-MD, 3851 Roger Brooke Drive, Fort Sam Houston, TX 78234-6200, USA. E-mail: adeangelo@pol.net white blood cell count remained normal. Blood cultures grew β-hemolytic group F streptococcus within 24 h (identified by Streptex® latex agglutination beads; Abbot Diagnostics, Abbot Park, IL). The patient was treated with ampicillin/ sulbactam and defervesced in 24 h. Follow-up blood cultures were negative. Antibiotics were continued for a total of 10 days to treat streptococcal bacteremia, and the patient recovered without further complications.

### DISCUSSION

To our knowledge, group F streptococcal bacteremia from a Bartholin's abscess has not been reported in the English-language literature. A review of this literature (MEDLINE) reported after 1966 revealed a paucity of cases of bacteremia from Bartholin's abscesses. Reported bloodstream pathogens in only two previous reports included *Peptostreptococcus* and *Propionibacterium* species[1] and *Escherichia coli*[2].

Lancefield and Hare[3] first described group F streptococci in 1928, and identification in the laboratory today still relies on specific reactivity with the Lancefield group F streptococcal antibody (in our case, a latex bead test). Several investigators have subsequently attempted to categorize this collection of organisms further[4–7]. Although a universally accepted taxonomy does not exist, group F streptococci are often included in the larger group, *Streptococcus milleri*, which also variably includes *S. anginosus, S. intermedius, S. constellatus*, and *S. MG. S. milleri* is considered by many to refer to a physiologically homogeneous species with antigenic and hemolytic heterogeneity[4]. All of the *S. milleri*-group streptococci, including group F strains, share the clinical characteristic of causing suppuration, an otherwise unusual feature of streptococcal infections.

Group F streptococci naturally occur in the oral cavity and the respiratory, gastrointestinal and urogenital tracts[4,8,9]. Despite being commonly isolated from the urogenital tract, particularly the vagina, group F streptococci are not commonly associated with specific infections at these sites[4,5]. Only one study has recorded the isolation of group F streptococci from Bartholin's abscesses (in two cases; no clinical data were presented)[8]. Group F streptococci have been associated with brain, dental and hepatic abscesses, occasional endocarditis and wound infections[4,5].

Group F streptococci are not a frequent cause of bacteremia. In a study of all bacteremias at Mayo Clinic-affiliated hospitals from 1970 to 1980, group F streptococci accounted for only 2% of all β-hemolytic streptococcal isolates[10]. In five of the seven cases in which these organisms were isolated, some form of manipulation or pathology was associated with the gastrointestinal tract, making it the most likely portal of entry. When other sources of group F streptococcus isolation not associated with bacteremia were scrutinized in the same study, the genitourinary tract was the most common deep site of origin, with vaginal and urine sources representing the majority. A recent review of 51 cases of bacteremia involving the *S. milleri* group reported the gastrointestinal and hepatobiliary tracts and the thoracic cavity as the most common sources of infection but did not record any cases related to Bartholin's gland cysts or abscesses[11].

Microbiologic evaluations of Bartholin's gland abscesses have revealed genitourinary tract anaerobes, Gram-negative bacilli, microacrophilic streptococci, enterococci and staphylococcal species as the predominant organisms[12,13]. A 10-year review of suppurative genitourinary tract infections reported the isolation of *S. intermedius* from one of 26 cases of Bartholin's gland abscesses, although this isolate was not specifically characterized as group F[12].

Currently, there are no data regarding the duration of treatment for group F streptococcal bacteremia. When an abscess is recognized and drained, there are no data to suggest that a course of treatment (intravenous or oral) should extend beyond 10–14 days. A longer course of treatment might be reasonable when the initial focus is not completely drained or when there is evidence of suppurative metastatic foci.

In summary, whereas group F streptococci are known to inhabit various body sites and have a predilection for forming abscesses, bacteremia from these organisms is infrequent. Being common colonizers, they have occasionally been identified in true infections of the genitourinary tract, but only very rarely in Bartholin's abscesses. This case of group F streptococcal bacteremia following self-drainage of a Bartholin's abscess constitutes the first such description in the medical literature.

### REFERENCES

1. Lopez-Zeno JA, Ross E, O'Grady JP. Septic shock complicating drainage of a Bartholin gland abscess. *Obstet Gynecol* 1990;76:915–16
2. Carson GD, Smith LP. *Escherichia coli* endotoxic shock complicating Bartholin's gland abscess. *Can Med Assoc J* 1980;122:1397–8
3. Lancefield RC, Hare R. The serologic differentiation of pathogenic and nonpathogenic strains of hemolytic streptococci from parturient women. *J Exp Med* 1935;61:335–49
4. Whitworth JM. Lancefield group F and related streptococci. *J Med Microbiol* 1990;33:135–51
5. Shlaes DM, Lerner PI, Wolinsky E, Gopalakrishna KV. Infections due to Lancefield group F and related streptococci (*S. milleri, S. anginosus*). *Medicine* 1981;60:197–207
6. Molina J-M, Leport C, Bure A, *et al.* Clinical and bacterial features of infections caused by *Streptococcus milleri*. *Scand J Infect Dis* 1991; 23:659–66
7. Facklam RR. The major differences in the American and British *Streptococcus* taxonomy schemes with special reference to *Streptococcus milleri*. *Eur J Clin Microbiol* 1984;3:91–3
8. Poole PM, Wilson G. Occurrence and cultural features of *Streptococcus milleri* in various body sites. *J Clin Pathol* 1979;32:764–8
9. Wort AJ. Observations on group F streptococci from human sources. *J Med Microbiol* 1975;8:455–7
10. Libertin CR, Hermans PE, Washington JA II. Beta-hemolytic group F streptococcal bacteremia: a study and review of the literature. *Rev Infect Dis* 1985;7:498–503
11. Bert F, Bariou-Lancelin M. Lambert-Zechovsky N. Clinical significance of bacteremia involving the "*Streptococcus milleri*" group: 51 cases and review. *Clin Infect Dis* 1998;27:385–7
12. Brook I. Anaerobic bacteria in suppurative genitourinary infections. *J Urol* 1989;141:889–93
13. Wren WD. Bacteriological findings in cultures of clinical material from Bartholin's abscess *J Clin Pathol* 1977;30:1025–7

Received 7/18/00; Accepted 11/17/00
